# Warm Ischemic Injury Is Reflected in the Release of Injury Markers during Cold Preservation of the Human Liver

**DOI:** 10.1371/journal.pone.0123421

**Published:** 2015-03-30

**Authors:** Bote G. Bruinsma, Wilson Wu, Sinan Ozer, Adam Farmer, James F. Markmann, Heidi Yeh, Korkut Uygun

**Affiliations:** 1 Center for Engineering in Medicine, Department of Surgery, Massachusetts General Hospital/ Harvard Medical School, Boston, Massachusetts, United States; 2 Department of Surgery (Surgical Laboratory), Academic Medical Center, University of Amsterdam, Amsterdam, the Netherlands; 3 Transplant Center, Massachusetts General Hospital/ Harvard Medical School, Boston, Massachusetts, United States; IDIBAPS—Hospital Clinic de Barcelona, SPAIN

## Abstract

**Background:**

Liver transplantation plays a pivotal role in the treatment of patients with end-stage liver disease. Despite excellent outcomes, the field is strained by a severe shortage of viable liver grafts. To meet high demands, attempts are made to increase the use of suboptimal livers by both pretransplant recovery and assessment of donor livers. Here we aim to assess hepatic injury in the measurement of routine markers in the post-ischemic flush effluent of discarded human liver with a wide warm ischemic range.

**Methods:**

Six human livers discarded for transplantation with variable warm and cold ischemia times were flushed at the end of preservation. The liver grafts were flushed with NaCl or Lactated Ringer’s, 2 L through the portal vein and 1 L through the hepatic artery. The vena caval effluent was sampled and analyzed for biochemical markers of injury; lactate dehydrogenase (LDH), alanine transaminase (ALT), and alkaline phosphatase (ALP). Liver tissue biopsies were analyzed for ATP content and histologically (H&E) examined.

**Results:**

The duration of warm ischemia in the six livers correlated significantly to the concentration of LDH, ALT, and ALP in the effluent from the portal vein flush. No correlation was found with cold ischemia time. Tissue ATP content at the end of preservation correlated very strongly with the concentration of ALP in the arterial effluent (P<0.0007, R^2^ = 0.96).

**Conclusion:**

Biochemical injury markers released during the cold preservation period were reflective of the duration of warm ischemic injury sustained prior to release of the markers, as well as the hepatic energy status. As such, assessment of the flush effluent at the end of cold preservation may be a useful tool in evaluating suboptimal livers prior to transplantation, particularly in situations with undeterminable ischemic durations.

## Introduction

Liver transplantation has become the mainstay treatment for end-stage liver disease and provides excellent outcomes [1]. Nevertheless, many thousands of deaths result from liver failure each year that could not be treated due to lack of suitable donor organs [2]. To better meet clinical demands, expanded use of livers donated after circulatory death (DCD) is being explored and their use has increased 10-fold in the United States [3]. However, increased injury to these organs during procurement and preservation and, consequently, a higher incidence of graft failure [4] and biliary complications [5] hinders realization of their full potential.

Pretransplant liver viability assessment remains of particular interest in subobtimal livers, including livers from DCD donors. Reliable and sensitive viability markers could allow the use of liver grafts that would otherwise be discarded. Novel *ex vivo* machine perfusion preservation methods are under investigation that not only appear to support and improve function[6,7], but importantly also enable functional assessment [8–10]. Alternatively, pre-transplant sampling of the effluent cold preservation solution may be a viable, simpler option. In a porcine model, easily measurable markers such as ammonia and lactate were significantly increased in the collected effluent and other studies have similarly identified potential indicators [11–14].

**Fig 1:**
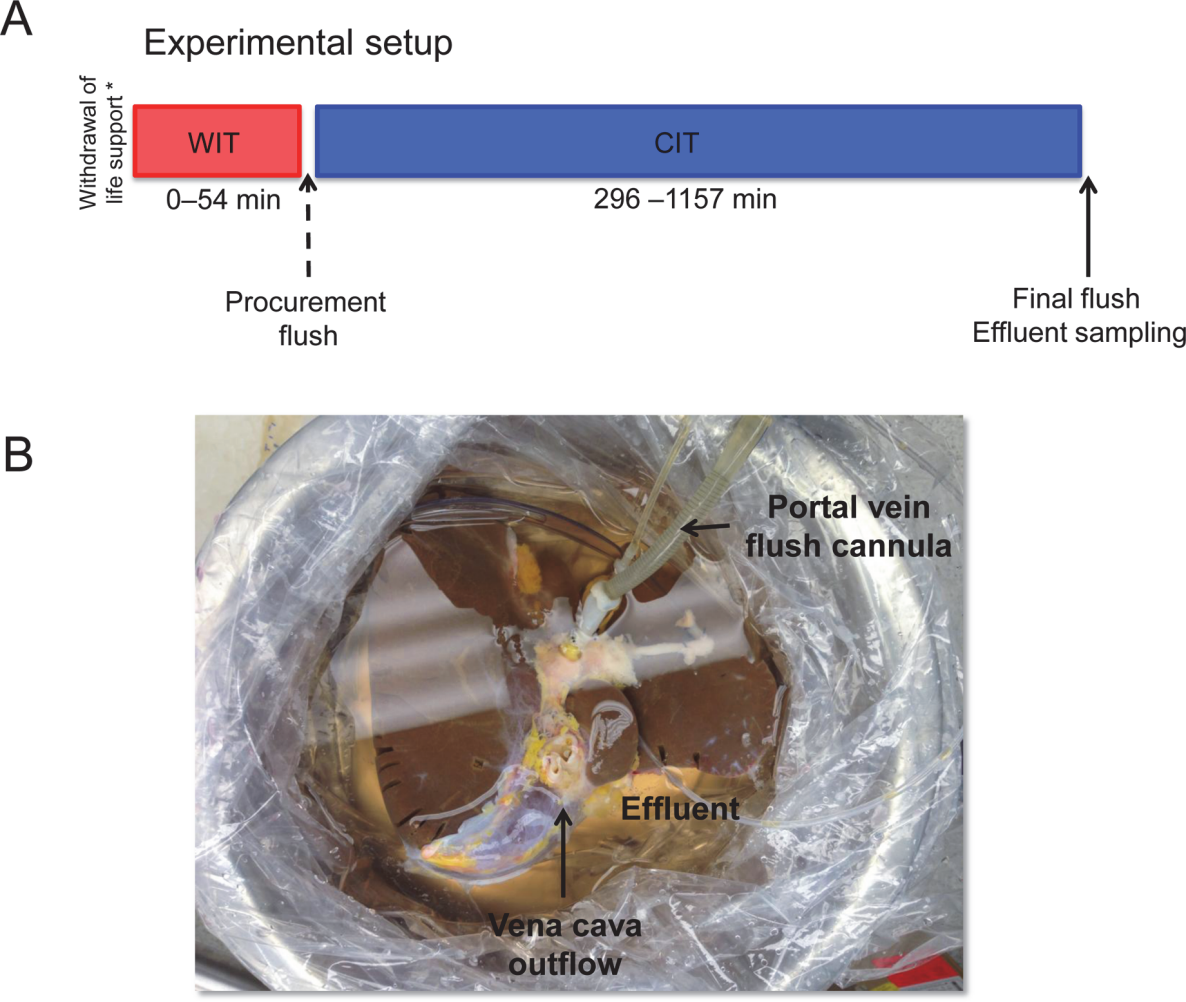
Experimental setup. Human livers are procured following standard liver procurement technique. Donation after circulatory death (DCD) livers were exposed to warm ischemic time ranging from 20–54 minutes. Following warm ischemia livers are abundantly flushed and stored in University of Wisconsin solution until arrival at our center. Livers are finally unpacked and flushed with 2 L of normal saline through the portal vein and 1 L through the hepatic artery (A), the effluent of which is sampled for analysis (B).

Injury markers released during warm ischemia are washed out of the liver during extensive flush on procurement, therefore sampling of the effluent at the end of preservation reflect injury markers released during cold ischemia (Fig 1A). Nevertheless, since preceding warm ischemic injury exacerbates cold preservation injury, we hypothesized that warm ischemia may be reflected in the post-cold preservation flush effluent. To investigate this, the effluent was analyzed for routine markers of hepatocellular injury and correlated to the duration of ischemic injury. Since warm ischemic durations currently tolerated clinically are limited (<30 min), we made use of human livers discarded for transplantation with a large variation in warm and cold ischemic times to assess the influence of both warm and cold ischemia.

## Materials and Methods

### Experimental study and human livers

Six human livers discarded for transplantation were donated for use in this work and obtained through and consented by the New England Organ Bank (NEOB). The use of discarded human livers was reviewed and declared exempt by the Massachusetts General Hospital Institutional Review Board (IRB No. 2011P001496). None of the transplant donors were from a vulnerable population and all donors or next of kin provided written informed consent that was freely given. Five livers were procured following circulatory death, while one liver was donated after brain death (DBD). Livers were procured following standard procurement technique, described in detail elsewhere [7]. Importantly, livers were flushed *in situ* with 4–6 L of UW solution through the aorta and additionally on the back table with an additional 2 liters through the portal vein, washing out injury markers released during warm ischemia. In the case of DCD donors, extubation and declaration of death (5 minutes after circulatory cessation) was performed by the primary service. Warm ischemia time (WIT) is defined as the time between extubation and start of *in situ* aortic cold flush in DCD donors and ranged from 20 to 54 minutes (Table 1). The DBD liver was procured without warm ischemia, with an *in situ* and back table flush similar to the DCD livers. The DBD liver was discarded for transplantation as a result of macrovesicular steatosis (20–30%) combined with donor age. All livers were cooled in ice-cold UW solution and packaged in sterile boxes for transportation. Cold ischemic time (CIT) ranged from 296 to 1157 minutes. On arrival at our center the vasculature of the liver was prepared for flushing by cannulating the portal vein and hepatic artery as described elsewhere [7]. The liver was first flushed through the PV with 2 L of NaCl or Lactated Ringer’s (LR) solution, after which the effluent was thoroughly mixed and sampled (Fig 1B). Next, the HA was flushed with 1 L of flush solution and the effluent sampled stored at −80°C. Parenchymal liver biopsies were taken and snap frozen in liquid nitrogen. Additional tissue biopsies of the liver were fixed in 10% buffered formalin.

**Table 1:** Pre-transplant and preservation parameters.

	**1**	**2**	**3**	**4**	**5**	**6**
**Donor type (DCD/DBD)**	DCD	DCD	DCD	DCD	DBD	DCD
**Gender**	M	M	M	F	M	M
**Age (yrs)**	25	50	50	62	75	51
**BMI**	26.7	24.6	25.8	30.18	26	26.5
**Cause of death**	Anoxia	Anoxia	Head trauma	Resp. failure	Head trauma	Anoxia
**Ischemia**						
WIT	23 min	54 min	28 min	34 min	N/A	20 min
CIT	1002 min	1157 min	296 min	740 min	685 min	525 min
**Macrosteatosis**	< 10%	< 10%	< 10%	< 10%	20–30%	< 10%

*DCD*, *donation after cardiac death; DBD*, *donation after brain death; WIT*, *warm ischemia time; CIT*, *cold ischemia time*

### Effluent and tissue analysis

The effluent was analyzed for concentrations of lactate dehydrogenase (LDH; BioVision Inc, Milpitas, CA), alkaline phosphatase (ALP; BioVision Inc, Milpitas, CA), alanine transaminase (ALT), and aspartate transaminase (AST) (InfinityTM ALT(GPT) and AST (GOT) liquid stable reagent kits Cellomics/Thermo Electron, Pittsburgh, PA, USA). Glucose was measured using a Rapidpoint 500 blood gas analyzer (Siemens, Norwood, MA). Adenosine triphosphate (ATP) was measured in liver tissue biopsies. Briefly, the tissue was pulverized while frozen in liquid nitrogen and assayed using a luminescence-based assay kit (BioVision Inc, Milpitas, CA, USA) and normalized to protein concentration determined using Coomassie dye. Formalin-fixed liver biopsies were subsequently stained with hematoxylin and eosin (H&E).

### Statistical analysis

Data were analyzed in Prism 5.0a for Mac OS X (GraphPad software, Inc., La Jolla, CA). Correlation between variables in human livers was assessed by linear regression, using a squared Pearson correlation coefficient (r^2^) as a measure of linear correlation. A p-value of less than 0.05 was considered statistically significant.

## Results

**Fig 2:**
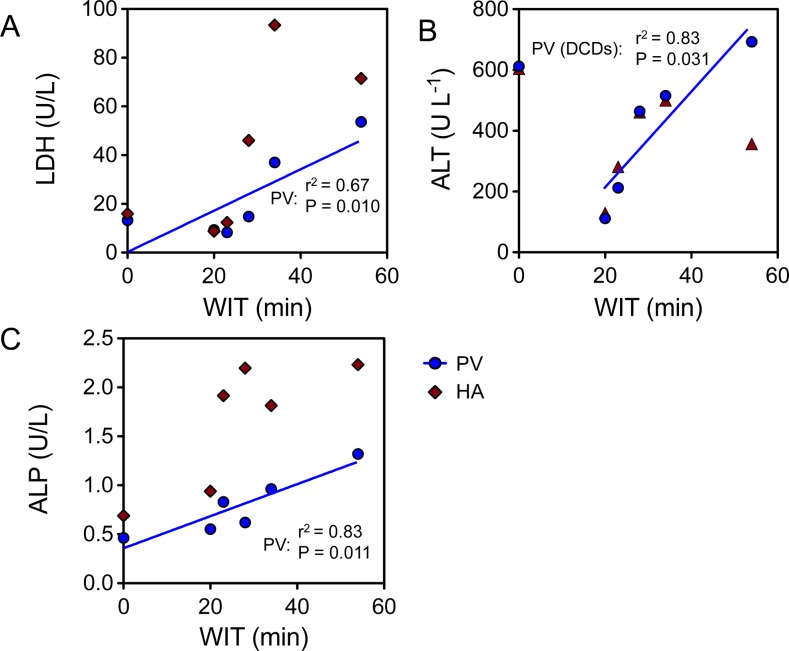
Effluent correlation with warm ischemic injury. Warm ischemic injury in the 6 grafts was correlated to the flush effluent concentration of biochemical markers of injury. WIT showed a positive correlation with the concentration of LDH (A), ALT (B), and ALP (C) in the effluent of the portal vein flush (blue line, P<0.05 for all). The correlation of ALT was strongest amongst DCD livers.

The enzyme release after preservation was quantified and correlated to duration of ischemia. The release of LDH into the preservation solution during cold preservation is shown in Fig 2A and ranged from 8.31 to 53.6 U/L (median = 14.0 U/L). Increasing WIT resulted in an increase in LDH activity in the effluent, resulting in a positive correlation between the two when measured in the effluent following 2 L of flush through the portal vein (r^2^ = 0.67, P = 0.048). The correlation was slightly stronger (r^2^ = 0.92, P = 0.010) amongst DCD livers, which could be attributed to relatively high release of LDH in the steatotic DBD graft (13.24 U/L). The release of ALT ranged from 111.7 to 692.44 U/L (Fig 2B), a correlation again was found between ALT activity in in the effluent following the PV flush in DCD livers (r^2^ = 0.83, P = 0.031). The second highest ALT activity was found in the effluent of the DBD liver (611.74 U/L). Effluent ALP activity correlated to WIT in all livers, with the least activity in the DBD liver (Fig 2C; r^2^ = 0.83, P = 0.011). Glucose release did not appear to depend on WIT in human livers, as glucose showed no clear correlation (not shown). No correlation was found between CIT and markers released in the effluent (P>0.05).

**Fig 3:**
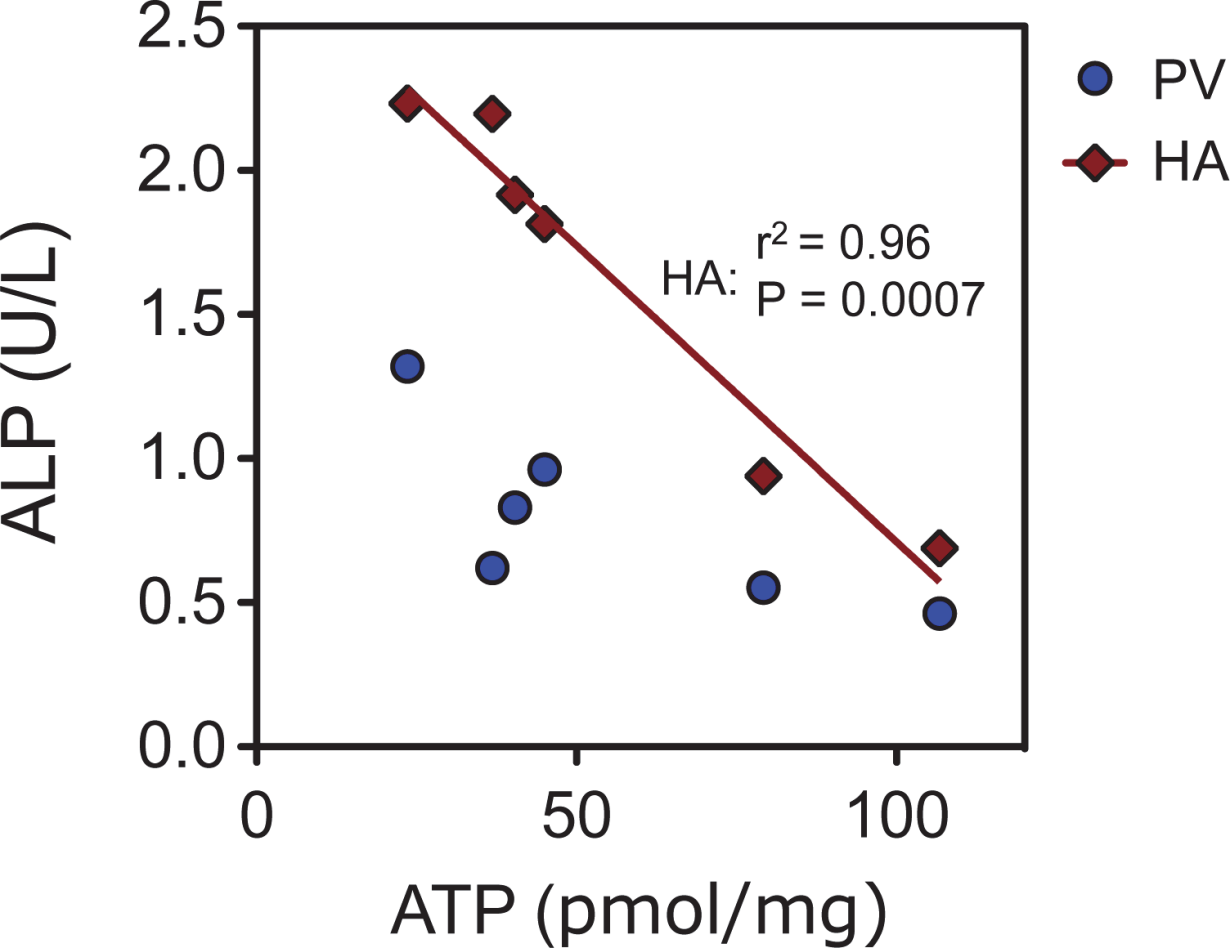
Effluent correlation with ATP. Tissue ATP content at the end of preservation showed a high correlation with the concentration of alkaline phosphatase in the flush effluent of the hepatic artery (red line, P<0.0007, R^2^ = 0.96).

**Fig 4:**
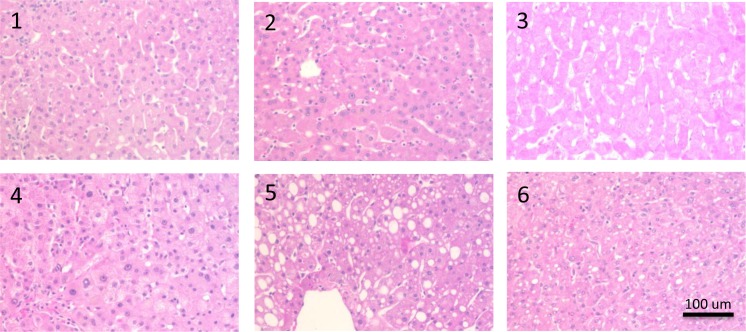
Post-preservation histology. Representative photomicrographs are shown for the 6 livers after H&E staining. No significant injury was observed after cold preservation, besides moderate macrosteatosis in graft #5.

ATP content ranged from 23.56–106.8 pmol/mg protein. While no association was found between ATP and ALT, LDH or glucose (p>0.05 in all cases), a strong correlation was observed between ATP and ALP released in the hepatic artery flush (Fig 3; R^2^ = 0.96, P = 0.0007). Since morphological injury mostly only occurs after reperfusion histological evaluation of the liver tissue revealed no significant injury and could not be quantified. Representative histology is shown in Fig 4.

## Discussion

During liver preservation, the cold preservation solution forms the direct interface with the organ. Cellular products are released during preservation and accumulate in the static solution. Here, we investigated whether measurement of the preservation solution effluent is indicative of the injury the liver sustained during procurement (warm ischemia) and preservation (cold ischemia) in discarded human livers. The human livers used in this study were considered to be of suboptimal viability for transplantation, the majority discarded as a result of warm ischemic injury on procurement. The wide range of warm ischemic times (20–54 minutes) made their use ideal for studying the correlation between warm ischemia and effluent release of potential indicators of injury. Although the release of LDH, ALT, and ALP occurred during cold preservation, no correlation was found between CIT and effluent concentrations. Interestingly, however, release of these enzymes in the flush effluent did increase with increasing warm ischemic time.

Current liver preservation cools the organ down, attenuating ischemia that would occur much more rapidly at normothermic temperature. In the DCD setting, cold preservation is inevitably preceded by a brief period of warm ischemia during procurement of the liver. While injurious in itself, it has been proposed that warm ischemia exacerbates injury sustained during subsequent cold ischemia. For instance, a porcine preservation study demonstrated that even a brief period of cold ischemia following warm ischemia could render a liver untransplantable [15]. In a clinical series, it was also found that warm ischemia increased the release of hepatocellular enzymes during cold preservation [16]. The number of DCD livers in the cohort was small, but findings corroborated what was found here in mostly DCD livers with a large range of WIT. These results suggest that sequential warm and cold ischemia synergistically injure the liver. Transplant outcomes further support this fact, as cold ischemia is one of the more important risk factors in DCD liver transplantation [17,18], with 60-day graft failure rates increasing from 10.8% to 58.3% with <8 hours and >12 hours of CIT, respectively [19]. The predictive potential of effluent analysis was suggested in an analysis by Devlin et al., where effluent AST activity was shown to correlate to post-operative AST, rejection rates and 1-month graft survival. Moreover, Schenk et al. used a compound score of effluent glutathione S-transferase, glutamate dehydrogenase and leucocyte count to predict graft survival with 95% accuracy [20]. Lange et al. found that LDH activities greater than 2-fold the median had a 53% greater risk of initial non-function and delayed graft function and all cases of graft dysfunction showed high effluent LDH concentrations[21]. A recent clinical series showed the potential value of flush analysis for biliary complcaitions. Low levels of cholangiocyte-derived microRNAs in the pre-transplant flush predicted the development of non-anastomotic biliary strictures [22].

Similar to our results, others have also found no significant correlation between the duration of cold ischemia—which ranged from 6–15 hours—and release of hepatocellular injury markers (LDH, AST and ALT)[16].

This study using discarded livers allowed for the unique assessment of the lives with a broad range of ischemic injury, offering a set of parameters that to our knowledge has not been studied before due to the limited inclusion of DCD livers in the clinical setting. The DCD livers did not have a level of steatosis, nor a high donor age considered deleterious to liver viability [23,24]. One of 6 livers studied in this series, was donated after brain death and discarded for excessive steatosis (30% macrovesicular) combined with donor age. It appears that the combination of age and steatosis increased the effluent concentration of ALT, in particular. It is to be expected that other (preoperative) parameters influence effluent enzyme release, but the effect of other factors is likely not conveyed. The use of effluent sampling presented here as a specific indicator of viability is diminished by unrepresented factors, including vascular/biliary injury to the graft, inflammatory activation and rejection from the recipient. Expanding these simple parameters to include biliary markers, including recently indentified microRNAs [22] would improve the evaluation of DCD grafts in particular. Sinusoidal endothelial cells appear particularly susceptible to cold preservation [25,26]. Indicators of endothelial damage, such as uptake of hyaluronic acid from the flush solution have been shown to reflect cold ischemic injury and may therefore be another valuable addition [27].

Various studies have demonstrated that ATP holds a strong correlation to ischemic injury and the importance of ATP for liver viability is strongly supported, where ATP depletion correlates well to both warm and cold ischemic injury [28,29], and may be predictive of transplant outcome [30,31]. A large porcine transplant model that showed that cold ischemic tolerance was strongly reduced by the duration of preceding warm ischemia, and importantly that this was reflected in ALT activity and ATP content [32]. ATP measurements are difficult to perform routinely and as a result surrogate markers that correlate to tissue ATP, such as ALP in this study, would be a valuable addition to the pretransplant assessment of suboptimal livers. Particularly in the case of uncontrolled DCD procurement where donor WIT is unknown, such measurements are of value in determining the degree of injury sustained. Moreover, predicting the ischemic severity remains difficult and multiple definitions of warm ischemia currently co-exist[33,34], beginning anywhere between withdrawal of life support and cardiac arrest. A more quantitative assessment of ischemic injury may prove invaluable in identifying optimal DCD donors [35].

## Conclusion

In conclusion, the markers studied here, despite being released during cold ischemia, correlate well to the severity of warm ischemic injury in human livers. As such, effluent sampling and analysis for various markers of cellular injury and ischemia appear to be a valuable tool for simple and fast assessment of graft injury in DCD liver transplantation.
